# Identification of Genetic Susceptibility Factors Associated with Canine Gastric Dilatation-Volvulus

**DOI:** 10.3390/genes11111313

**Published:** 2020-11-05

**Authors:** Ignazio S. Piras, Nieves Perdigones, Victoria Zismann, Natalia Briones, Salvatore Facista, José Luis Rivera, Elizabeth Rozanski, Cheryl A. London, William P. D. Hendricks

**Affiliations:** 1 Neurogenomics Division, Translational Genomics Research Institute, Phoenix, AZ 85004, USA; ipiras@tgen.org; 2 Integrated Cancer Genomics Division, Translational Genomics Research Institute, Phoenix, AZ 85004, USA; nievich@gmail.com (N.P.); vzismann@tgen.org (V.Z.); nbriones@tgen.org (N.B.); sfacista@tgen.org (S.F.); jriver32@asu.edu (J.L.R.); 3 Cummings School of Veterinary Medicine, Tufts University, Grafton, MA 01536, USA; elizabeth.rozanski@tutfs.edu (E.R.); Cheryl.London@tufts.edu (C.A.L.)

**Keywords:** gastric dilatation volvulus, bloat, Genome-Wide Association Study

## Abstract

Canine gastric dilatation-volvulus (GDV) is a common life-threatening condition occurring primarily in large and giant breeds with a 3.9% to 36.7% lifetime risk. The genetic correlates of GDV have not previously been systematically explored. We undertook an inter-breed genome-wide association analysis (GWAS) of 253 dogs from ten breeds including 106 healthy dogs and 147 dogs with at least one GDV episode. SNP array genotyping followed by imputation was conducted on 241 samples to identify GDV-associated single-nucleotide polymorphisms (SNPs) and copy number variations (CNVs). A subset of 33 dogs (15 healthy dogs and 18 GDV patients from the three most represented breeds) was characterized by whole genome sequencing (WGS). After genome-wide Bonferroni correction, we identified a significant putatively protective intergenic SNP (rs851737064) across all breeds. The signal was most significant in Collies, German Shorthaired Pointers, and Great Danes. Subsequent focused analysis across these three breeds identified 12 significant additional putatively protective or deleterious SNPs. Notable significant SNPs included those occurring in genes involved in gastric tone and motility including *VHL, NALCN,* and *PRKCZ*. These data provide important new clues to canine GDV risk factors and facilitate generation of hypotheses regarding the genetic and molecular underpinnings this syndrome.

## 1. Introduction

Gastric dilatation-volvulus (GDV or bloat) is a life-threatening condition in dogs that presents at a frequency of 2.9 to 6.8 per 1000 canine hospital admissions [1] as sudden intragastric pressure followed by stomach dilation and rotation. The compression of major abdominal vessels due to gastric torsion leads to impaired cardiac output and shock within hours, requiring emergency stabilization and surgical correction, followed by gastropexy securing the stomach to the abdominal wall. Without corrective surgery (gastropexy), GDV recurs in up to 80% of affected dogs (median overall survival of 188 days [2]), compared to a recurrence of less than 5% with gastropexy [2,3,4]. Importantly, approximately 44% of GDV patients seeking emergency care are euthanized. Prophylactic gastropexy may decrease the prevalence of GDV in susceptible breeds, but it has associated risks such as frequent mesenteric volvulus [5]. Therefore, better prophylactic strategies guided by improved understanding of risk factors and development of new diagnostics are urgently needed.

GDV is common in dogs [3], but rare in humans and uncommon in other species [6,7,8,9,10,11,12]. Breed is the most significant risk factor associated with GDV in dogs, with those most commonly affected including Great Danes (14.0% prevalence), Akitas (9.2%), Dogue de Bordeaux (7.2%), Irish Setters, and Weimaraners (7.1%) [3]. Odds ratios (OR) for some of the most common breeds include Great Danes, Weimeraners, Saint Bernards, Gordon Setters, and Irish setters are 10.0, 4.6, 4.2, 4.1, and 3.5, respectively [1]. Mixed-breed dogs are affected as well, but their risk is lower than for Great Dane, Saint Bernard, Weimaraner, Irish Setter, Gordon Setter Standard Poodle, Basset Hound, Doberman Pinscher, Old English Sheepdog, and German Shorthaired Pointer breeds [1]. Overall, mixed-breed dogs account for 6.0% to 15.7% of GDV affected dogs [13]. Increased body weight is associated with increased risk [1] as well as lean body condition [14] and increased thoracic-depth-to-width ratio [1,15,16]. Other risk factors in dogs include primary ligament laxity [17], dysregulation of gastric motility (abnormal gastric motility patterns, delayed gastric emptying, and, sporadic acute gastric dilation) [2], and chronic gastric instability (CGI) which often precedes GDV episodes [18,19]. Various dietary factors are also associated with GDV risk such as smaller food size particles in Great Danes [20], dry foods containing an oil or fat among the first 4 ingredients [21], feeding a single food type, and a large volume of food at a time [14]. Lastly, family history of GDV (OR: 1.90), and age (OR 1.15) are also significant risk factors. Together, these data suggest the existence of a genetic component of GDV risk [22].

Although few studies have explored the genetic basis of GDV, it has been hypothesized that genetic susceptibility factors associated with GDV may be immune-derived [23]. This hypothesis is based on the co-occurrence of canine GDV with inflammatory bowel disease (IBD), and the consideration that the immune system may be modulating the gastrointestinal (GI) microbiome, autoimmunity, or both. In human IBD cases, alterations in GI tract tone and motility have been observed alongside delayed gastric emptying [24] and gastroparesis [25]. In targeted studies of immune-associated genes including: *DLA88* exons 2 and 3, *DRB1* exon 2, *TLR5* exon 1, *NOD2* exon 3, and *ATG16L1* exon 8, a significant association of TLR5*B (an allele defined by T8A changes in TLR5) with GDV was identified in great Danes, with a prevalence of 19% in GDV cases and 7% in controls (OR, 3.10; *p* = 0.007) [23]. The study was conducted by characterizing 39 dogs with at least one GDV episode and 42 controls. The TLR5*B allele encodes for one of the two variants at the amino acid level (the alternative being TLR5*A), characterized by 4 SNPs including the T8A, associated with IBD (inflammatory bowel disease) [26,27]. The same group also found differences in the microbiome composition of GDV-affected and unaffected dogs [28]. However, no study has yet systematically assessed the genetic susceptibility factors of GDV. Here, we used multi-platform genomic analysis (SNP array and whole genome sequencing) to identify GDV genetic susceptibility factors in 10 breeds. We identified 27 significant independent SNPs putatively associated with GDV risk. Notably, 11 of these regions occur within or near genes associated with central roles in GI tone/motility.

## 2. Materials and Methods

Samples were collected from 253 dogs treated at the Cummings Veterinary Medical Center Henry and Lois Foster Hospital for Small Animals as well as from select local emergency clinics and specialty hospitals. All cases had radiographic confirmation of GDV (inclusive of a right lateral radiograph presenting a classic “Smurf sign”) and underwent subsequent surgical correction with diagnostic confirmation. For non-Tufts cases, in addition to blood samples, owners/veterinarians sent medical confirmation of GDV (radiographs, surgical reports, and associated clinical data). Dogs defined as chronic bloaters were those that continued to have bloat based on follow-up despite gastropexy, suggestive of ongoing gastrointestinal issues. The majority of healthy controls (HC) were collected via breed clubs and dog shows. Healthy controls were senior dogs (>8 years of age) confirmed to have never had a preventative gastropexy or an episode of GDV. Given that some studies have shown GDV incidence to increase with age, it remains possible that those dogs still alive may yet experience GDV. For those cases for which family history was available, these data were collected from breeders at specialty shows with knowledge of parents and littermates. The cohort consisted of 147 GDV patients and 106 healthy controls (HC) across 13 breeds. Sample collection occurred under supervision of the attending veterinarian following institutional approvals (Tufts approved protocol CSRC#041-13 on July 2nd, 2014) and informed consent from the pet owner. Peripheral blood samples were collected from all animals with DNA extracted by Qiagen DNeasy Blood and Tissue kit or Qiagen Genomic DNA Midi Kit (Qiagen Inc., Hilden, Germany # 69504, # 51183). The DNA was quantified by Qubit with the Qubit Broad Range dsDNA assay kit (Life Technologies, Carlsbad, CA, USA #Q32853). The DNA quality was determined by DNA Integrity Number on an Agilent 4400 Tapestation (Agilent, Santa Clara, CA, USA #G2991AA).

SNP array genotyping was performed on 253 samples using the Illumina Canine HD Whole-Genome Genotyping Bead Chip arrays including 173,662 SNPs located on autosomes and sex chromosomes. The genotype calling of SNPs as well as data visualization were performed with *GenomeStudio v2.0* (Illumina, San Diego, CA, USA) and exported to *PLINK* [29] format using the *CanFam3.1.75* genome assembly [30] to retrieve chromosomal coordinates. SNP array data is available in the Gene Expression Omnibus repository, accession GSE141128.

Whole Genome Sequencing (WGS) was conducted on a subsample of 33 dogs (18 GDV cases and 15 HC) from the three most prevalent breeds (Borzoi, Great Dane, and GSD). Informatic tools, versions, and flags are shown in Table S1. Whole genome libraries were sequenced on the Illumina NovaSeq 6000 producing paired end reads of 150 nucleotides. FASTQ files were aligned to the canine reference genome (*CanFam3.1.75*) using BWA v0.7.8 [31]. Binary Alignment Map (BAM) files were realigned and recalibrated using *Genome Analysis Toolkit* (*GATK*) *v3.3.0* [32] and duplicate pairs were marked with *Picard v1.128* (http://broadinstitute.github.io/picard). Custom scripts were used for genomic variant calling per chromosome and joint variant calling per multiple samples. Germline SNVs were called using *HaplotypeCaller* (GATK v3.8.1.0) and *SAMtools v1.9*. The resulting gvcf files were used as input to do joint variant calling for the cases and controls within each of the three breeds with *GenotypeGVCFs* and default parameters from *GATK v3.8.1.0.* The resulting vcf file was filtered using *VCFtools v1.017* [33] including only “PASS” single nucleotide variants with not more than 2 alleles, and using the following thresholds: depth ≥5, quality ≥300, genotype quality (GQ) ≥20, and SNP genotyping rate ≥95%. The filtered vcf files were converted to *PLINK* format [29] for downstream analysis. WGS BAMs, corresponding index files, and metadata are available in the NCBI Sequence Read Archive (Submission SUB6265963).

SNP array data imputation was conducted using a phased genotype reference dataset of 365 dogs from multiple breeds genotyped by whole-genome sequencing [34]. The reference dataset was filtered excluding sex chromosomes, removing insertion/deletions, non-PASS variants, and variants with more than two alleles. The SNP array dataset of all 241 dogs on our study (target dataset) was filtered excluding sex chromosomes and duplicate variants, including variants with: minor allele frequency (MAF) ≥ 0.005, genotyping rate ≥95%, Hardy–Weinberg *p*-value in unaffected: >1.0 × 10^−7^, and samples with genotyping rate ≥90%. The resulting dataset was subjected to principal component analysis (PCA) in *PLINK v1.9* to identify outliers and dogs whose PCA clustering would differ from the owner-identified breed information. We computed the relatedness PIHAT metric using *PLINK v1.9* for each pair of samples aiming to identify duplicated samples (PIHAT = 1.0). Outliers, duplicated, and misclassified samples were removed from the dataset. Reference and target datasets were compared using the Conform-gt software (https://faculty.washington.edu/browning/conform-gt.html), keeping only variants shared between the target and the reference dataset. Finally, the genotypes were imputed with the software Beagle v5 [35], using the recombination rate map reference [36], default settings and effective population size = 200. After imputation, variants with dosage R^2^ (DR^2^) < 0.4 were filtered out, ultimately including only those with MAF ≥ 0.05, genotyping rate ≥ 95%, Hardy–Weinberg *p*-value in unaffected: >1.0 × 10^−7^, and samples with genotyping rate ≥90%. The gene annotation of the SNPs was performed with *R-BiomartT v2.40.4* [37] using the dog genome reference *CanFam3.1* [30].

Allele frequencies were compared with WGS data from Plassais et al. [38], including 722 dogs from several pure and mixed breeds. The data were downloaded from ncbi.nlm.nih.gov/sra; Bioproject number: PRJNA448733, and filtered using *vcftools* removing INDELs and with the following thresholds: depth ≥5, quality ≥300, and genotype quality (GQ) ≥30. We obtained a total of 16,761,109 variants. Of these, 212 variants overlapped with the significant SNPs we obtained in the GWAS. Allele frequencies were computed using *vcftools.*

CNV analysis was conducted on the SNP array data using *PENNCNV* [39]. The signal intensity file, including B-allele frequency (BAF) and log-R ratio (LRR), was exported using *Genome Studio*, and filtered including only informative and quality-control checked SNPs and samples. Additionally, samples with LRR standard deviation ≥0.3 were removed. The PBF (population allele-b frequency) file was generated from the signal file using the command *compile_pfb.pl*. The GC model file was created using the UCSC model file *canFam3.gc5BaseMOD.wig* using the function *cal_gc_snp.pl,* aiming to conduct GC signal correction during the CNV calls. Finally, the CNV calls were conducted using the *detect_cnv.pl* command. The CNV raw file was then filtered keeping CNV called by at least 5 consecutive SNP signals, and converted to the PLINK format using the command *penncnv_to_plink.pl*. The association analysis affected vs. unaffected (AF vs. UF) was conducted using PLINK with 50,000 permutation testing.

GWAS analysis was conducted applying the same workflow for both WGS and SNP array imputed data. We used a Mixed Linear Model as implemented in the *GEMMA v0.9* software [40], adjusting for relatedness, population substructure, and sex. In the first step of the analysis the algorithm estimates a centered relatedness matrix [40] used in the second step to adjust for sample structure after eigen-decomposition. The significance was assessed with the Wald test, and *p*-values were adjusted for multiple testing using the Bonferroni method accounting for the number of independent SNPs according to the Linkage Disequilibrium (LD) estimated with the command --*indep 100 10 10* as implemented in *PLINK v1.9*. Specifically, 100 is the window size in kb where the LD is computed, 10 is the number of variants the windows is shifted, and 10 is the inflation factor computed as 1/(1 − r^2^), corresponding to r^2^ = 0.9. Only SNPs with adjusted *p* < 0.05 were considered as significant. Quantile-quantile plots (Q-Q plots) and λ inflation factor were computed using the R-package *snpStats*. The lambda inflation factor (computed as the ratio of the median of the empirically observed distribution of the test statistic to the expected median) quantifies the extent of the bulk inflation and the excess false positive rate. A value of λ < 1.1 was considered as a condition to define the absence of significant population stratification. Usually, λ around 1.05 is considered an acceptable value, as reported in some previous large canine GWAS [41,42,43]. Manhattan plots for the relevant signals were generated using a custom R script, including the regional LD computed as pairwise R^2^ between the peak SNP and all the other variants located in the range ±250,000 bp from the peak SNP.

The concordance rate between WGS and SNP array experiments (including also imputed SNPs) was estimated using the overlapping samples genotyped with both technologies (12 cases and 9 HC from Borzoi, German Shepard Dog, and Great Dane breeds). The comparison was conducted by SNP genomic position using the filtered datasets (see previous “data imputation” and “Whole Genome Sequencing” methods paragraphs) as implemented in PLINK v1.9 software, specifically using the *--bmerge* function with *--merge-mode 7* option.

## 3. Results

### 3.1. Cohort Characteristics

To identify genetic risk factors associated with canine GDV, we undertook a multi-platform genomic analysis of 147 GDV cases collected at Tufts Cummings Veterinary Medical Center and other local veterinary clinics, and 106 healthy controls (HC, *n* = 253) collected through breed groups and dog shows. To reduce diversity, we removed samples belonging to breeds with 5 or fewer samples. The use of a larger cutoff would have dramatically decreased the total sample size and thus the power to detect associated variants. To account for the heterogeneity across the several breeds included in the study, the GWAS was adjusted for population structure and relatedness computing and including a kinship matrix in the model. The cohort subjected to SNP array analysis included 140 GDV cases (136 presenting in the acute form and 4 chronically) and 101 HC, 52 of which had no recorded family history of GDV and were considered Super Controls (SC). We also included the 4 cases of chronic GDV to increase power. The samples were inspected after genetic analysis by PCA to identify outliers, breed misclassification, and duplicates. A total of 215 dogs passed quality control filters, including 125 GDV cases and 90 HC, 45 of which were SC. This final cohort included 10 breeds, primarily Borzoi (25.6%), GSD (21.9%), and Great Dane (13.0%). The other breeds were Standard Poodle (9.8%), Doberman Pinscher (6.0%), Briard (5.6%), Labrador Retriever (4.7%), Golden Retriever and German Shorthaired Pointer (both 4.7%), and Smooth Collie (4.2%). The cohort was sex-balanced (51.0% male) and primarily neutered/spayed (66.0%). Although limitations in the availability of confirmed breed-matched healthy controls conferred an imbalance in the distribution of breeds between cases and controls in this cohort, simulation analyses (see Hayward et al. [43]) have supported that inter-breed GWAS, even when breed-imbalanced, may still have the power to detect causal loci. For WGS analysis, 33 dogs (21 of which were also analyzed by SNP array) were analyzed that were equally distributed across Borzoi, Great Dane, and GSD breeds (18 GDV cases and 15 HC, 7 of which were SC), as well as also gender and neutered balanced (51.5% female, 54% neutered/spayed). All samples genotyped by WGS passed quality control filters. An extended clinical annotation is shown in Table S2 and Figure S1. A summary of the sample size and informative SNPs used for the GWAS is reported in Table 1.

### 3.2. Identification of 26 Independent SNPs Associated with GDV through GWAS and Imputation from SNP Array Analysis 

Through SNP array genotyping, a total of 173,662 SNPs in 140 GDV cases and 101 HC were identified. After quality control filtering, two samples were removed for low genotype rate (<90%) and an additional 24 samples were removed for breed misclassification or because they were duplicates or outliers. The final cohort included 125 GDV cases and 90 HCs (45 SC) with 122,961 (total cohort) and 147,197 (SC cohort) informative SNPs. The SNP density and the sensitivity of our GDV GWAS study was then increased by inferring unobserved genotypes through imputation analysis using a reference canine dataset specifically selected and tested for imputation consisting of a total of 365 samples from multiple breeds genotyped with WGS [34]. Imputation was conducted separately in the case-HC (*n* = 215) and case-SC (*n* = 170) datasets because of the different number of informative SNPs available. In the total case-HC cohort, 5,593,011 high quality imputed SNPs (DR^2^ ≥ 0.4) were obtained. After applying standard filters (MAF, genotyping rate, Hardy–Weinberg) the number was reduced to 4,139,410 (399,810 independent). In the case-SC cohort, the imputation yielded 5,776,849 high quality variants (DR^2^ ≥ 0.4), which, after standard filtering, was reduced to 4,519,848. Altogether, the imputation allowed us to run the GWAS with around three times more genetic variants than with the SNP array platform alone.

**Figure 1:**
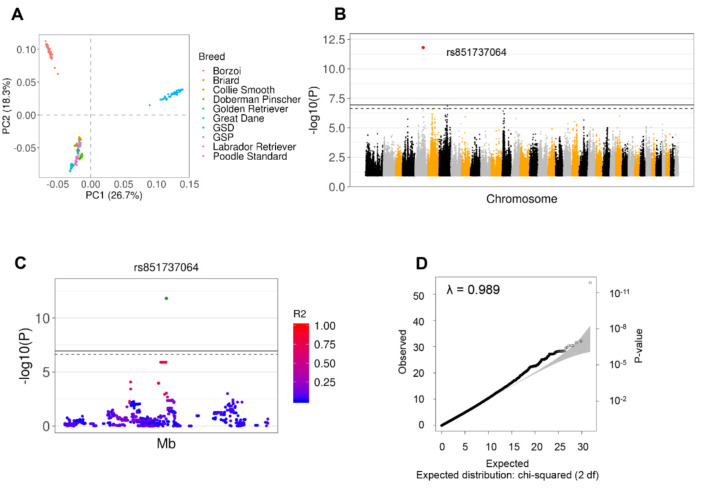
Identification of gastric dilatation-volvulus (GDV)-Associated SNPs based on GWAS from SNP Array data across all Breeds. (**A**) Scatterplot showing breed distribution using the two most informative Principal Components for the entire cohort, including all controls. (**B**) Manhattan plot for the SC cohort after adjusting for sex. A significant signal was detected across all breeds for the SNP rs851737064, indicated in red, using both the total and SC cohorts. The colors indicate different chromosomes. (**C**) Details of the rs851737064 region (±250,000 bp) showing the LD expressed as R^2^ between rs851737064 (green dot) and all other SNPs in the region. (**D**) A Q-Q plot showing correlation between expected and observed test statistic distribution. The inflation factor showed absence of population stratification (λ = 0.989).

After performing GWAS in the total cohort, adjusting for relatedness, population structure, sex, and multiple testing (accounting for 399,810 independent SNPs), a single significant signal for a protective allele at rs851737064 on chr5:57,632,433 (β = −0.488; adj *p* = 3.9 × 10^−2^) was identified. The significance for the same variant increased when the SC samples were used (adjustment for 434,516 SNPs: β = −0.564; adjusted *p*-value = 6.8 × 10^−7^). In both results, the minor allele G had a protective effect with a prevalence of 1.2% in GDV cases and 20% in SC (Figure 1 and Table 2, Tables S3 and S4). The same variants showed a larger allele frequency (7%) in the data from Plassais et al. [38].

**Figure 2:**
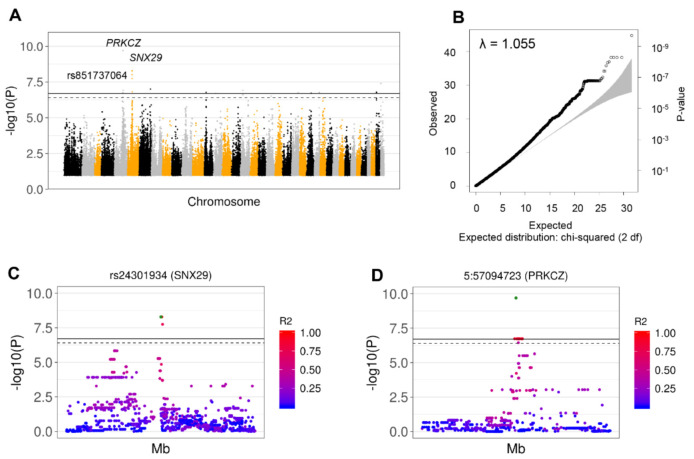
Identification of GDV-Associated SNPs based on GWAS from SNP Array Data in Collies, German Shorthaired Pointers, and Great Danes. Significant SNPs were identified by GWAS across these breeds when excluding controls with GDV family history. (**A**) Manhattan plot for the SC cohort after adjusting for sex showing significant SNPs in Collies, Great Danes, and German Shorthaired Pointers. The colors indicate different chromosomes. (**B**) A Q-Q plot showing the correlation between expected and observed test statistic distribution. The inflation factor showed absence of population stratification (λ = 1.055). (**C**) Details of the top SNP 5:57094723 detected in the *PRKCZ* region (±250,000 bp) showing the LD expressed as pairwise R^2^ between the top SNP (green dot) and all the other SNPs in the region. (**D**) Details of the top SNP rs24301934 detected in the *SNX29* region (±250,000 bp) showing the LD expressed as pairwise R^2^ between the top SNP (green dot) and all the other SNPs in the region.

After genotype and allele frequency inspection, the protective signal was found to be associated with Collies, German Shorthaired Pointers, and Great Danes. In order to confirm this observation, we then performed GWAS using only these three breeds (29 GDV cases and 18 HC) analyzing 4,107,331 informative SNPs. We detected a total of 26 significant SNPs after Bonferroni adjustment for 255,266 independent SNPs (adj *p* < 0.05), with the major allele always associated with higher risk (top SNP frequency: 94.8% in AF, 55.6% in UF). The signal for rs851737064 remained significant in the total cohort as well as the SC cohort (adj *p* < 0.01). Additional significant SNPs specific for these three breeds were found in *PRKCZ*, *SNX29*, *ERC2*, *PRKG1*, *TXNDC11*, and 10 chromosomal intergenic regions (Figure 2, Table 1, Tables S5 and S6).

Since 3 breeds showed a large imbalance between cases and controls (Golden Retrievers: only cases; Labrador Retrievers: 8 cases and 2 controls; Standard Poodle: 20 cases and 1 controls), we re-ran the GWAS excluding these breeds to assess whether the lack of balance was driving the association. When we used all the controls (with GDV family history and non, for a total of 87 cases and 87 controls), we did not detect significant associations (Table S7). However, the SNP rs851737064 (significant in the main analysis with adj-*p* < 0.05) was ranked among the top SNPs (29th and 8th in the analysis non-adjusting and adjusting for sex, respectively) in the GWAS conducted excluding the controls with GDV family history (87 cases and 43 controls) (Table S8). We compared the effect sizes (β) between the total analysis and after removing the 3 breeds, and we always obtained a significant and positive correlation (*ρ*> 0.882; *p* < 2.2 × 10^−16^).

**Figure 3:**
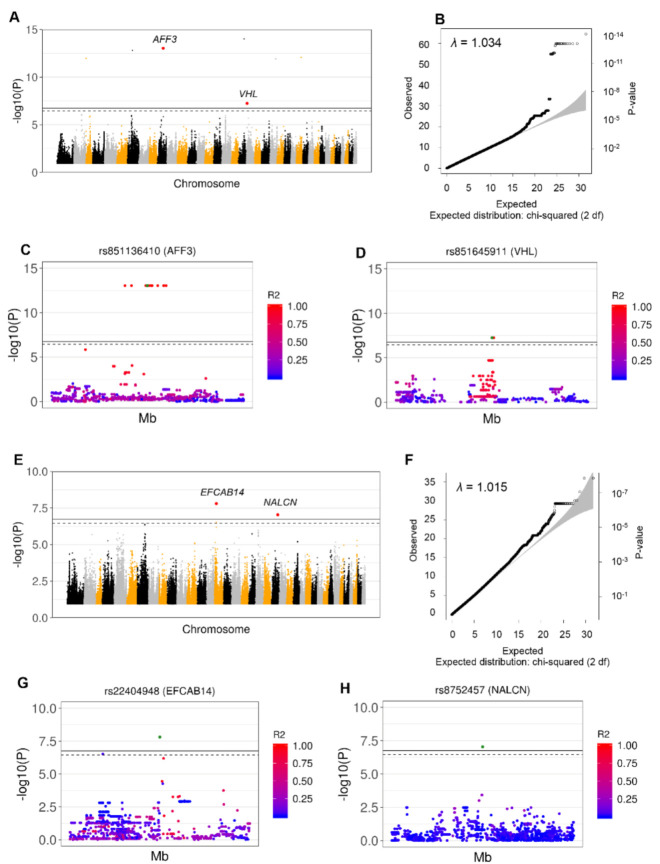
Identification of GDV-Associated SNPs based on GWAS from WGS Data in Borzoi, Great Danes and GSDs. Significant SNPs were identified by GWAS across these breeds when including all samples (A to D) and excluding controls with GDV family history (E to H). (**A**) Significant signals detected adjusting for sex. Top SNPs for each peak are highlighted in red—chromosome in 10 (rs851136410; *AFF3*) and chromosome 20 (rs852404461; *VHL*). The colors indicate different chromosomes. (**B**) A Q-Q plot showing the correlation between expected and observed test statistic distribution. The inflation factor showed absence of population stratification (λ = 1.034). (**C**) Details of the rs851136410 (*AFF3)* region (±250,000 bp) showing the LD expressed as R^2^ between rs851136410 (green dot) and all other SNPs in the region. (**D**) Details of the rs851645911 (*VHL*) region (±250,000 bp) showing LD expressed as R^2^ between rs851645911 (green dot) and all other SNPs in the region. (**E**) Significant signals detected adjusting for sex. The top SNPs for each peak are highlighted in red – chromosome 15 (rs22404947; *EFCAB14*) and chromosome 22 (rs8752457; *NALCN*). The colors indicate different chromosomes. (**F**) A Q-Q plot showing the correlation between expected and observed test statistic distribution. The inflation factor showed absence of population stratification (λ = 1.015). (**G**) Details of the rs22404947 (*EFCAB14*) region (±250,000 bp) showing the LD expressed as R^2^ between rs22404947 (green dot) and all the other SNPs in the region. (**H**) Details of the rs8752457 (*NALCN*) region (±250,000 bp) showing the LD expressed as R^2^ between rs8752457 (green dot) and all the other SNPs in the region.

Using the same imputed data, signals across the three most prevalent breeds in our cohort (Borzoi, Great Dane, and GSD) were investigated. GWAS was undertaken using 4,093,737 SNPs in the total case-HC cohort (69 GDV cases and 61 HC), and 4,283,353 informative SNP in the case-SC cohort (69 GDV cases and 25 SC). In the total cohort we identified 24 significant SNPs (correction for 274,818 SNPs) in chromosome 10 within or near the *AFF3* gene (adj *p* = 4.20 × 10^−10^) and this was also confirmed following adjustment for sex. The major frequency alleles in all SNPs were deleterious (frequency top SNP: 89.9% in AF, 37.3% in UF). In this cohort another signal in the sex-adjusted GWAS was found within the *VHL* gene (adj *p* = 1.58 × 10^−2^). In the SC cohort significant signals were discovered in the genes *EFCAB14* (adj *p* value < 0.01) and *NALCN* (adj *p* value < 0.05). In both cases a protective effect of the minor alleles was observed (Table 1, Figure 3, Tables S9 and S10).

Finally, we sought to identify breed-specific signals within the most prevalent breeds in our study (Borzoi, Great Dane, and GSD). In the Borzoi SC cohort consisting of 10 GDV cases and 19 SC, 76 significant SNPs were observed, all located on Chromosome 22, encompassing two large main regions at chr22:31,667,799-31,690,760, and chr22:32,424,536-32,460,177. These regions do not contain any known genes (Table 1, Figure S2 and Table S11). We did not detect any significant SNPs when we considered the other two breeds. The previously reported GDV-associated T8A variant in TLR5 could not be definitively interrogated in our cohort due to low or absent coverage in WGS and imputed data. 

### 3.3. Nine Independent SNPs Associated with GDV Were Identified by GWAS from Whole Genome Sequencing Data

WGS was performed using DNA from 18 GDV cases and 15 HC (seven of which were considered SCs) that included Borzoi, GSD, and Great Dane breeds. This yielded 17,832,385 total variants, with 12,969,066 “PASS” single nucleotide variants containing not more than 2 alleles. After pre-filtering (quality, depth, and GQ) 3,267,709 variants were obtained across all samples. The average sequencing coverage was 35.6X (range: 16.7 X–92.0 X), with a median of 3,264,020 variants/sample (range: 3,216,829–3,266,596). The number of informative SNPs in the total cohort after filtering for MAF, Hardy–Weinberg, and SNP genotyping rate cutoffs as above was reduced to 2,837,247. After adjusting for multiple testing and sex the final number of SNPs in the SC sample was 2,794,769, accounting for 198,090 independent signals. These data yielded 6 significant independent SNPs that were all located in chr7:60392183-60392380 (containing no known genes), and another 3 SNPs in chromosomes 7:29089260-29091216, within the *SLC19A2* gene (Table 1, Figure S3, Table S12). In all cases the major alleles were associated with higher risk of GDV. The λ showed some extent of population stratification for the sex-adjusted analysis (λ = 1.170), but not for the sex-unadjusted analysis (λ = 1.018), where the signal in the region chr7:60392183-60392380 was confirmed. No significant signals were detected in the total cohort. The discrepancy with the microarray data is possibly a consequence of the lower sample size and of the non-complete overlap between the two cohorts (only 21 overlapping samples).

All regions showing significant signals in microarray or WGS GWAS, but not including known dog genes, were further investigated for orthologues genes in *Homo sapiens*, *Mus musculus,* and *Rattus norvegicus* using the UCSC genome browser. However, the genes occurring in these regions in other species do not appear to be functionally relevant for the GDV genotype (Table S13).

### 3.4. Concordance of Imputation and WGS Data

**Figure 4:**
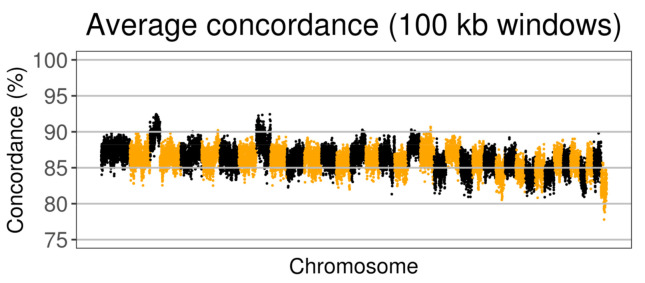
Concordance Between SNP Array Imputed Data and WGS Data. Concordance rates were computed using 1,737,178 overlapping SNPs and 21 samples characterized with both platforms, counting the number of concordant genotypes normalized by sample size in non-overlapping windows of 100 kb. The colors indicate different chromosomes.

Using the overlapping samples from 12 cases and 9 HCs from the three most common breeds in our study (Borzoi, Great Dane, and GSD), the concordance between SNP array imputed and WGS data was evaluated, demonstrating 1,737,178 overlapping SNPs with a concordance rate of 86.6%. Figure 4 shows the concordance (%) across all the genome averaged for 100 kb windows. 

Interestingly, this concordance rate is quite similar with the chromosome average imputation accuracy found by Hayward et al. [34], who developed the reference panel (average: 89.1%; range: 84.3–93.5%). Additionally, we also found a lower concordance rate in smaller chromosomes, due to lower recombination rates. The concordance of SNP array imputed and WGS data for each genetic region significantly associated with GDV was explored, finding 100% concordance for the signals observed in *PRKCZ*, *VHL*, *NALCN*, and the signal on chromosome 22 affecting Borzois (with 12, 28, 14 and 10 alleles available for comparison, in 13, 5, 1, and 12 SNPs, respectively) (Table 1). However, the difference in signals between WGS and microarray analysis might be due to the incompletely overlapping cohorts analyzed.

### 3.5. Identification of CNVs Associated with GDV Risk

CNV analysis of SNP array data was conducted both across all breeds and also focusing only on Borzoi, Great Dane, and GSD together. A summary of results is reported in Table S14. A significant CNV association was found using all breeds, but not confirmed when the SC cohort was used. Specifically, a duplication in chr26:31,496,517-31,576,417 in 22.7% of GDV cases and 2.5% of unaffected controls (adj *p* < 0.05) was identified associated with GDV risk, located in a region lacking known genes. The only proximal gene based on cross-species sequence alignment was Tm2d1 (*R. norvegicus*) (Figure S4A). Focusing on Borzoi, Great Dane, and GSD, a deletion more frequent in UF (30.9%) than in AF dogs (4.5%) (adj *p* < 0.001) was found, encompassing the region chr33:2748039-2917476, a region without known canine genes or genes in other species. This CNV was present in both cohorts (all controls and only SC) (Figure S4B). We also identified a CNV (duplicated in most of AF) encompassing the region chr5:78223252-78265466, with a prevalence of 34.8% in AD and 10.9% in UF (adj *p* < 0.05) (Figure S4C). This result was not confirmed in the SC cohort. The analysis conducted on Borzoi, Great Dane, and German Shepard dogs as independent breeds did not yield any significant results. No significant CNVs were detected in any of the three breeds together in the WGS data.

## 4. Discussion

GDV is a common disease associated with deep-chested large-breed dogs. Data suggest that despite the contribution of environmental factors, genetics may also play a role in its development. Through multiplatform genomic analysis of 147 GDV cases and 106 unaffected controls from 10 different breeds, we identified GDV-associated SNPs that provide clues to GDV’s molecular underpinnings. We controlled for population stratification due to the large number of breeds, relatedness and previous family history of GDV in the cohort. The cohort included 125 GDV cases and 90 healthy controls, 45 of which had no previous family history of GDV and were considered Super Controls (SCs). The inherent breed imbalance between cases and unaffected controls in this cohort prevented us from identifying specific breed-associated variants. However, simulation analysis has shown in such cases that inter-breed GWAS can still have sufficient power to detect causal loci [43]. In keeping with this finding, lambda inflation factor values in our study overall supported the absence of significant bias in the results.

SNP array data imputation of the entire case-HC dataset allowed us to conduct the GWAS with 4,139,410 informative SNPs for the entire case-HC dataset and 4,519,848 SNPs in the SC cohort. We detected a candidate significant protective SNP in an intergenic region on chromosome five associated with GDV in all breeds (rs851737064, adjusted *p*-value = 9.9 × 10^–2^ and adjusted *p*-value = 6.8 × 10^−7^ when compared to SCs, both adjusted for sex). The frequency of the minor allele (G) was significantly higher in controls (20%) than in affected GDV patients (1.2%) and was restricted to Collie, German Shorthaired Pointer, and Great Dane breeds. GDV patients and controls from the remaining breeds were homozygous for the alternative allele and therefore not protected by this variant. The results were significant in the SC group after removing the three most imbalanced breeds between cases and controls (Golden Retrievers, Labrador Retrievers, and Standard Poodles). The allele frequency of rs851737064 in the data from Plassais et al. [38] was larger than in our GDV cases. Additionally, candidate GDV-associated variants in intergenic regions identified in this study included two CNVs—a duplication on chromosome 26 and a deletion on chromosome 33. In each of these cases, additional validation studies as well as exploration of underlying sequence variants and possible functional effects of these variants are warranted.

Although further studies are needed in order to validate the SNPs identified in this study and their association with GDV susceptibility (as well as potential breed associations), the genes in which they are located have notable roles in enteric nervous system and GI motility. For example, the investigation of GDV-associated SNPs in the rs851737064 susceptible breeds (Collies, GSD, and GD) unveiled an additional association with GDV only 0.5 Mb downstream of the SNP previously identified. The new association expanded a region of 20kb partially within the gene *PRKCZ* (protein kinase C zeta) and included 17 variants, three of which were SNPs (rs24231711, rs24220585, and rs24237788). Variants in the *PRKCZ* gene were also detected when we removed the imbalanced breeds. Moreover, the allele frequencies of these SNPs in the data from Plassais et al. [38] were larger than in our GDV cases. PRKCZ is a protein kinase C (PKC) serine/threonine kinase. Smooth muscles express such PKC isozymes, with roles in contraction and motility. The PKC-Z protein is expressed in the cytosol of smooth muscle cells of the antrum in rats [44]. Defective PKC-Z activation contributes to skeletal muscle insulin resistance and alteration of gastric smooth muscle contraction in diabetes and diabetes model systems, potentially in association with gastroparesis, which is common in diabetic patients [45,46,47]. PRKCZ is also a susceptibility factor for Type 2 diabetes. PRKCZ also plays a role in the development of the enteric nervous system [48] and interacts with VHL [49], another gene bearing significant GDV-associated SNPs in this study in Borzoi, Great Dane, and GSD breeds. Only Great Danes bore both susceptibility factors, SNPs within *PRCKZ* and *VHL*, in our cohort. Half of these GDV patients were homozygous for both susceptibility factors, with only 16% of healthy controls showing the same genotype (*p*-value not significant). 

We also investigated the presence of genetic risk factors in the three most common breeds in our cohort (Borzoi, GSD, and Great Dane). After adjustment for sex, we detected a total of 39 SNPs located in 7 different regions, 2 of them encompassing the genes *AFF3* (AF4/FMR2 Family Member 3) and *VHL* (von Hippel–Lindau tumor suppressor). The other five regions were located in gene deserts. Additionally, after correction for sex, we detected two more independent signals in the SC cohort located in in *EFCAB14* (EF-Hand Calcium Binding Domain 14) and *NALCN* (Sodium Leak Channel, Non-Selective). The comparison between imputed and WGS data for the overlapping samples showed 100% of concordance between the genotypes of the SNPs detected in *PRCKZ*, *VHL*, and *NALCN*. As with PRKZC, VHL plays important roles in GI tract motility regulation. *VHL* encodes a member of a multi-protein complex that regulates hypoxia-inducible factor 1 (HIF1) [50,51]. When VHL is dysregulated, HIF1 accumulates (as it does under hypoxic conditions) and binds to hypoxia-response elements (HREs) in the gastrin promoter leading to increases in gastrin expression. Gastrin stimulates secretion of gastric acid (HCl) and plays a role in gastric motility. Interestingly, plasma levels of gastrin are significantly increased in GDV patients in the fasted state following surgical intervention [52]. HIF1 accumulation also impacts serotonin and somatostatin levels [53], both with relevant roles in GI motility [54,55]. Thus, through its involvement in essential neuromodulators and GI tract regulatory pathways, VHL may play a role in GDV risk. *NALCN* encodes a sodium leak channel involved in GI motility through regulation of the intestinal pacemaking activity in the interstitial cells of Cajal [56] (ICC) located throughout the digestive system. They are part of the GI neuromuscular apparatus that transduces input from enteric motor neurons, generates intrinsic electrical rhythmicity, and has a mechanosensation role. The dysregulation of these cells has been linked to GI tract disorders including gastroparesis, a condition that affects the ability of the stomach to empty its contents and that presents without physical obstruction [57,58]. In normal conditions, antral muscle stretching due to food intake increases the slow wave frequency of ICCs by a non-neural mechanism. The response to gastric stretch is absent in muscles of mice lacking intramuscular ICC cells (ICC-IM). Pathogenic *NALCN* variants in humans are associated with severe hypotonia, speech impairment, cognitive delay [59], chronic constipation, and facial dysmorphism reminiscent of infantile hypotonia with psychomotor retardation and characteristic faces (IHPRF, OMIM #615419). Further, a case of delayed gastric emptying has also been described in a patient with variants in the channelosome gene, *UNC80* [60], an NALCN interactor in ICCs. Thus, *NALCN* is an additional compelling candidate GDV-associated gene given its role in GI motility and ICCs and as well as its role in behavioral disorders such as hyperactivity.

With respect to other potential candidate genes linked to GDV, the WGS analysis of Borzoi, Great Danes, and GSDs revealed three significant GDV-associated SNPs on chromosome 7 (rs2461000, rs24436905 and rs24436906). These polymorphisms are located in an intergenic region close to *CDH2* (Cadherin 2). Polymorphisms in CDH2 have been associated with canine obsessive-compulsive disorder [61]. Notably, anxious temperament is a risk factor for GDV occurrence in high-risk breeds. Finally, breed-specific associations of Borzoi with GDV were identified in this study, all involving RBM26 (RNA Binding Motif Protein 26) of unknown significance. Genetic variants in this gene were associated with Coal Workers’ Pneumoconiosis [62].

## 5. Conclusions

Through application of multi-platform genotyping methods and imputation analysis, we identified multiple cross-breed and breed-specific SNPs associated with canine GDV protection and pathogenicity. Importantly, our findings highlight the potential role of gastric tone and motility pathways mediated by PRKCZ, VHL, and NALCN. Further validation of these imputed SNPs and analysis of underlying sequence variants in additional canine breeds in larger cohorts is necessary to define their specific contribution to GDV risk, biology and pathology.

## Figures and Tables

**Table 1:** Summary of the cohorts used for the genome-wide association analysis (GWAS), including samples and single nucleotide polymorphisms SNPs passing quality control steps.

Breed	Technology	AF	UF	Informative SNPs	Independent SNPs **
Borzoi, Great Dane, GSD	WGS	18	15	2,837,247	209,534
Borzoi, Great Dane, GSD *	WGS	18	7	2,794,769	198,090
Borzoi, Great Dane, GSD	Imputed microarrays	69	61	4,093,737	274,818
Borzoi, Great Dane, GSD *	Imputed microarrays	69	25	4,283,353	282,309
All Breeds	Imputed microarrays	125	90	4,139,410	399,810
All Breeds *	Imputed microarrays	125	45	4,519,848	434,516
Borzoi	Imputed microarrays	10	45	3,335,814	141,714
Borzoi *	Imputed microarrays	10	19	3,405,912	140,326
Great Dane	Imputed microarrays	22	6	3,612,725	164,347
GSD	Imputed microarrays	37	10	3,231,787	119,747
Collie, GSP, Great Dane,	Imputed microarrays	29	18	4,057,970	255,266
Collie, GSP, Great Dane *	Imputed microarrays	29	14	4,107,331	253,661

* Includes controls without GDV family history. ** Number of independent SNPs according regional LD patterns used for the multiple test adjustment of GWAS *p*-values. GSD: German Shepherd; GSP: German Shorthaired Pointer. AF: affected; UF: unaffected; WGS: whole genome sequencing.

**Table 2:** Significant SNPs associated with Canine GDV across all breeds.

All Breeds	**Chr**	**bp**	**SNP ID**	**A1**	**A2**	**Gene**	**Entire Cohort**		**SC cohort**		Concordance (Allelic Count) **
							**Adj p**	Model *	**Adj p**	Model *	
	5	57632433	rs851737064	G	A	-	3.9 × 10^−2^	B	6.8 × 10^−7^	A,B	-
	5	63538480	-	C	T	-	-	-	-	-	-
	7	43676279	-	A	G	-	-	-	2.1 × 10^−2^	A	0.800 (20)
	7	43680845	-	A	G	-	-	-	2.1 × 10^−2^	A	0.800 (20)
	7	43691688	-	T	A	-	-	-	2.1 × 10^−2^	A	0.800 (20)
	7	43691953	-	T	C	-	-	-	2.1 × 10^−2^	A	0.800 (20)
	7	43678127	-	C	T	-	-	-	3.2 × 10^−2^	A	-
	5	47125974	-	A	G	-	-	-	4.6 × 10^−2^	B	-
Collie, GSD, GD	5	57094723	-	G	A	-	3.6 × 10^−3^	A,B	3.1 × 10^−5^	A,B	-
	5	57632433	-	G	A	-	7.2 × 10^−3^	A,B	1.3 × 10^−3^	A,B	-
	38	2356822	-	A	G	-	8.2 × 10^−3^	A,B	6.6 × 10^−3^	A,B	0.833 (12)
	6	29829315	rs24300758	C	A	-	1.2 × 10^−2^	A,B	1.3 × 10^−3^	A,B	0.833 (12)
	5	57091335	rs24231711	G	C	*PRKCZ*	3.4 × 10^−2^	A,B	2.0 × 10^−2^	A,B	1.000 (12)
	5	57097255	-	T	C	-	3.4 × 10^−2^	A,B	2.0 × 10^−2^	A,B	1.000 (12)
	5	57097774	-	T	C	-	3.4 × 10^−2^	A,B	2.0 × 10^−2^	A,B	1.000 (12)
	5	57099671	rs24220585	T	G	*C1orf86-PRKCZ*	3.4 × 10^−2^	A,B	2.0 × 10^−2^	A,B	1.000 (12)
	5	57099975	-	T	G	-	3.4 × 10^−2^	A,B	2.0 × 10^−2^	A,B	1.000 (12)
	5	57100286	-	G	T	-	3.4 × 10^−2^	A,B	2.0 × 10^−2^	A,B	1.000 (12)
Borzoi, GSD, GD	15	13750111	rs22404948	G	A	*EFCAB14*	-	-	4.0 × 10^−3^	A,B	0.800 (10)
	15	13750230	rs22404947	A	G	*EFCAB14*	-	-	4.0 × 10^−3^	A,B	0.800 (10)
	22	50824204	rs8752457	T	C	*NALCN*	-	-	2.6 × 10^−2^	A,B	1.000 (14)
	19	39848326	-	C	A	-	2.6 × 10^−9^	A,B	-	-	0.560 (16)
	10	42896897	-	C	T	-	2.6 × 10^−8^	A,B	-	-	-
	10	42913666	-	T	A	-	2.6 × 10^−8^	A,B	-	-	0.961 (26)
	10	42950857	-	G	T	-	2.6 × 10^−8^	A,B	-	-	0.961 (26)
	10	42954164	-	G	T	-	2.6 × 10^−8^	A,B	-	-	0.961 (26)
	10	42954515	rs851136410	T	C	*AFF3*	2.6 × 10^−8^	A,B	-	-	0.961 (26)
	10	42954869	-	T	C	-	2.6 × 10^−8^	A,B	-	-	0.961 (26)
Borzoi	22	31689520	-	G	C	-	-	-	6.1 × 10^−8^	A,B	0.800 (10)
	22	32134106	rs23055590	C	G	-	-	-	2.4 × 10^−5^	A,B	0.800 (10)
	22	32134940	rs23005916	A	G	-	-	-	2.4 × 10^−5^	A,B	0.700 (10)
	22	33041052	rs23063720	C	T	-	-	-	2.4 × 10^−5^	A,B	0.900 (10)
	22	33045940	-	G	T	-	-	-	2.4 × 10^−5^	A,B	0.900 (10)
	22	32424536	-	A	G	-	-	-	3.0 × 10^−5^	A,B	1.000 (10)
	22	32424591	-	C	T	-	-	-	3.0 × 10^−5^	A,B	1.000 (10)
	22	32424650	-	G	A	-	-	-	3.0 × 10^−5^	A,B	1.000 (10)
	22	32425463	rs23040592	C	T	-	-	-	3.0 × 10^−5^	A,B	0.800 (10)
	22	32453864	-	C	T	-	-	-	3.0 × 10^−5^	A,B	1.000 (10)
Borzoi, GSD, GD (WGS) ***	7	60392183	rs24461000	A	G	-	-	-	2.1 × 10^−2^	A,B	-
	7	60392379	rs24436905	C	T	-	-	-	2.1 × 10^−2^	A,B	-
	7	60392380	rs24436906	A	G	-	-	-	2.1 × 10^−2^	A,B	-
	7	29089260	-	A	G	-	-	-	1.1 × 10^−2^	B	-
	7	29090352	rs24427856	A	G	*SLC19A2*	-	-	1.1 × 10^−2^	B	-
	7	29091216	-	A	G	-	-	-	1.1 × 10^−2^	B	-

A1: Minor frequency allele; A2: Major frequency allele; “GSD” = German Shepherd; “GD” = Great Dane; * Model A and B: with and without sex as covariate; ** Comparison between WGS and SNP array (imputed) data for the samples characterized with both platforms. *** Lambda values for the B model suggested population stratification (lambda > 1.1).

## Supplementary Materials

The following are available online at https://www.mdpi.com/2073-4425/11/11/1313/s1, Figure S1: Extended Clinical Annotation, Figure S2: Identification of GDV-Associated SNPs based on GWAS from SNP Array Data in Borzoi after Adjusting for GDV Family History. (A) Significant signals detected in chromosome 22 after adjusting for sex. (B) A Q-Q plot showing the correlation between expected and observed test statistic distribution. The inflation factor showed absence of population stratification (λ = 1.055). (C) and (D) Details of the two most significant regions: chr22:31667799-31690760, and chr22:32424536-32460177. The LD is shown as pairwise R^2^ between the top SNPs (22:31689520 and rs23005916) and all other SNPs in the regions., Figure S3: Identification of GDV-Associated SNPs based on GWAS from WGS Data in Borzoi, GSDs, and Great Danes Adjusting for GDV Family History. (A) Scatterplot showing the breed distribution using the two most informative Principal Components for the entire cohort, including all controls. (B) Manhattan plots after adjusting for sex, showing the two significant signals. (C) A Q-Q plot showing the correlation between expected and observed test statistic distribution. The inflation factor showed some extent of population stratification (λ = 1.170), but not in the analysis non-adjusted for sex (λ = 1.018), where only the signal in the rs24436905 was detected. (D) Regional plot surrounding the significant SNP rs24427856 located in SLC19A2. The LD is shown as pairwise R^2^ between the top SNPs (rs24427856) and all other SNPs in the regions. (E) Regional plot surrounding the significant SNP rs24436905. The LD is shown as pairwise R^2^ between the top SNPs (rs24436905) and all other SNPs in the regions, Figure S4. Relevant results obtained with the CNV analysis using the microarray data. (A) All breeds: region located in chr26:31,496,517-31,576,417 including a duplication with frequency significantly higher in AF than UF (*p* < 0.05). The finding was not confirmed when we use the SC cohort. (B) Borzoi, Great Dane and GSD: region located in chr33:2748039-2917476 including a deletion significantly more frequent in UF than AF (*p* = 0.001). The finding was confirmed when we used the SC cohort, Table S1: Informatic tools, versions and flags, Table S2: Extended clinical annotation of samples passing quality filters, Table S3: Results from SNP Array imputation: top 25 SNPs detected in the GWAS using all breeds, Table S4: Results from SNP Array imputation: top SNPs detected in the GWAS (all breeds) after excluding controls with family history of GDV, Table S5: Results from SNP Array imputation: top SNPs in Collie, German Shorthaired Pointer, and Great Dane, Table S6: Results from SNP Array imputation: top 25 SNPs detected in the GWAS using Collie, German Shorthaired Pointer, and Great Dane without controls with known family history of GDV, Table S7: Results from SNP Array Imputation: Top 50 SNPs detected in the GWAS excluding the 3 breeds with large unbalance between cases and controls: Golden Retrievers, Labrador Retrievers, and Standard Poodle. Controls with and without family history of GDV were included (GDV = 87; HC = 87). We indicated in grey the SNP detected in the GWAS conducted using all breeds, Table S8: Results from SNP Array Imputation: top 50 SNPs detected in the GWAS excluding the 3 breeds with large unbalance between cases and controls: Golden Retrievers, Labrador Retrievers, and Standard Poodles. Only Controls with family history of GDV were included (GDV = 87; HC = 43). We indicated in grey the SNPs detected in the GWAS conducted using all breeds, Table S9: Results from SNP Array imputation: top 40 SNPs detected for Borzoi, German Shepard Dog, and Great Dane, Table S10: Results from SNP Array imputation: significant SNPs detected in Borzoi, German Shepard Dog, and Great Dane after excluding controls with family history of GDV, Table S11: Results from SNP Array imputation: significant SNPs detected in Borzoi removing controls with known family history of GDV, Table S12: Results from WGS analysis: significant SNPs detected after excluding controls with known family history of GDV, Table S13: Regions including significant SNPs located in dogs “gene deserts” investigated for orthologs genes in Homo sapiens (Hs), Mus musculus (Mm) and Rattus norvegicus (Rn), Table S14. CNV significantly different between affected and unaffected; A) All breeds including controls with and without GDV family history (GDV = 125; HC = 90); B) Borzoi, Great Dane, and German Shepherd Dog breeds including controls with and without GDV family history (GDV = 69; HC = 61); C) Borzoi, Great Dane, and German Shepherd Dog breeds including controls without GDV family history (GDV = 69; HC = 25).
