# Supplementary material for: Identification of Genetic Susceptibility Factors Associated with Canine Gastric Dilatation-Volvulus
Source: Genes (Basel). 2020 Nov 5;11(11):1313. doi: 10.3390/genes11111313 (PMC7694454; doi:10.3390/genes11111313)
Supplement: Supplementary file 1 [file genes-11-01313-s001.zip › supplementary figures.docx]

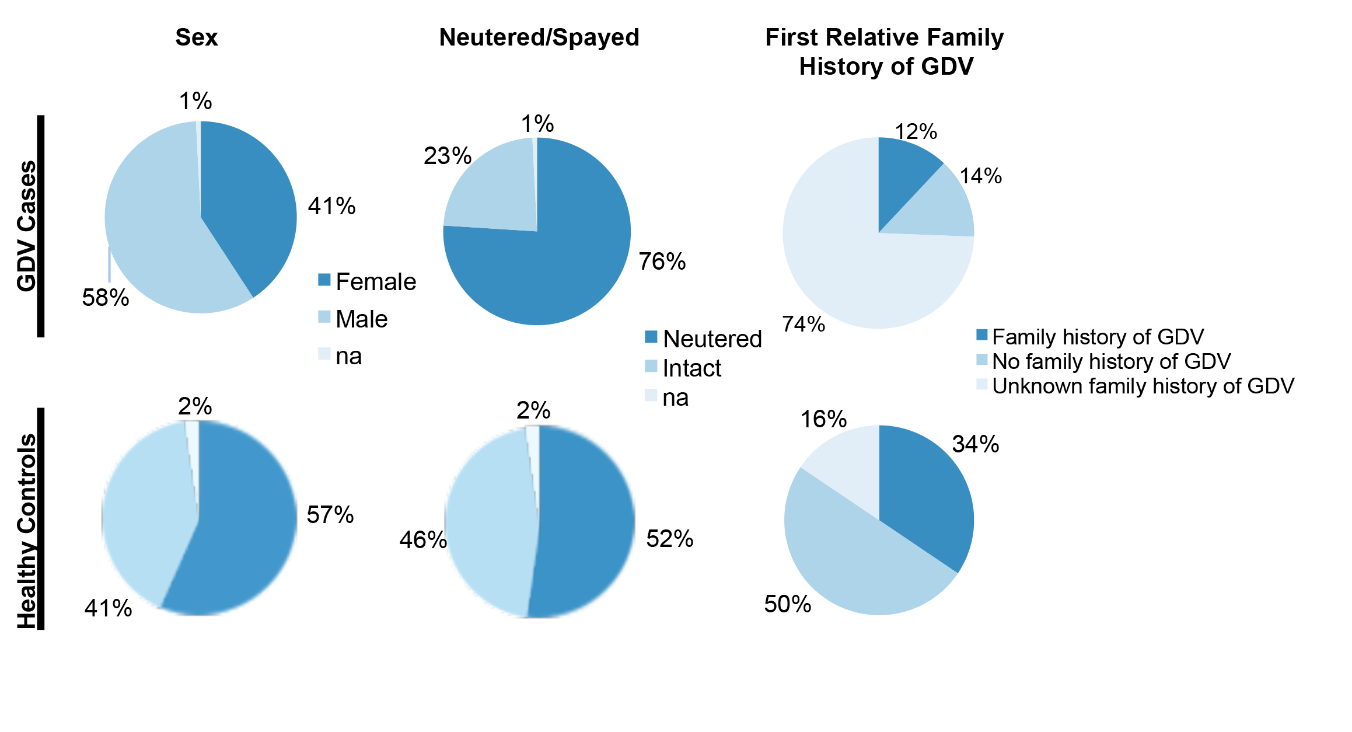


**Figure S1.** Extended Clinical Annotation.


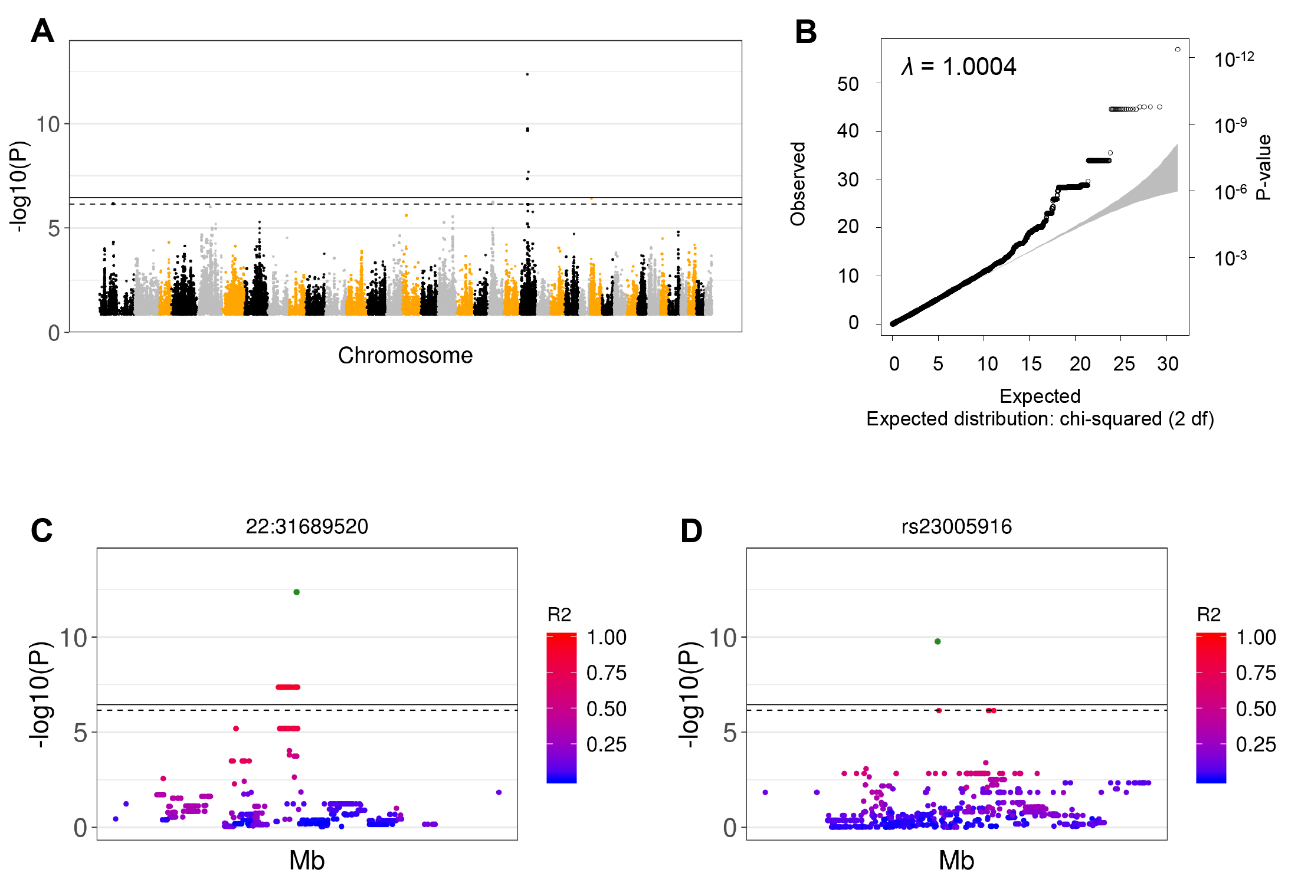


**Figure S2**: Identification of GDV-Associated SNPs based on GWAS from SNP Array Data in Borzoi after Adjusting for GDV Family History. (A) Significant signals detected in chromosome 22 after adjusting for sex. (B) A qqplot showing the correlation between expected and observed test statistic distribution. The inflation factor showed absence of population stratification (λ = 1.055). (C) and (D) Details of the two most significant regions: chr22:31667799-31690760, and chr22:32424536-32460177. The LD is shown as pairwise R^2^ between the top SNPs (22:31689520 and rs23005916) and all other SNPs in the regions.


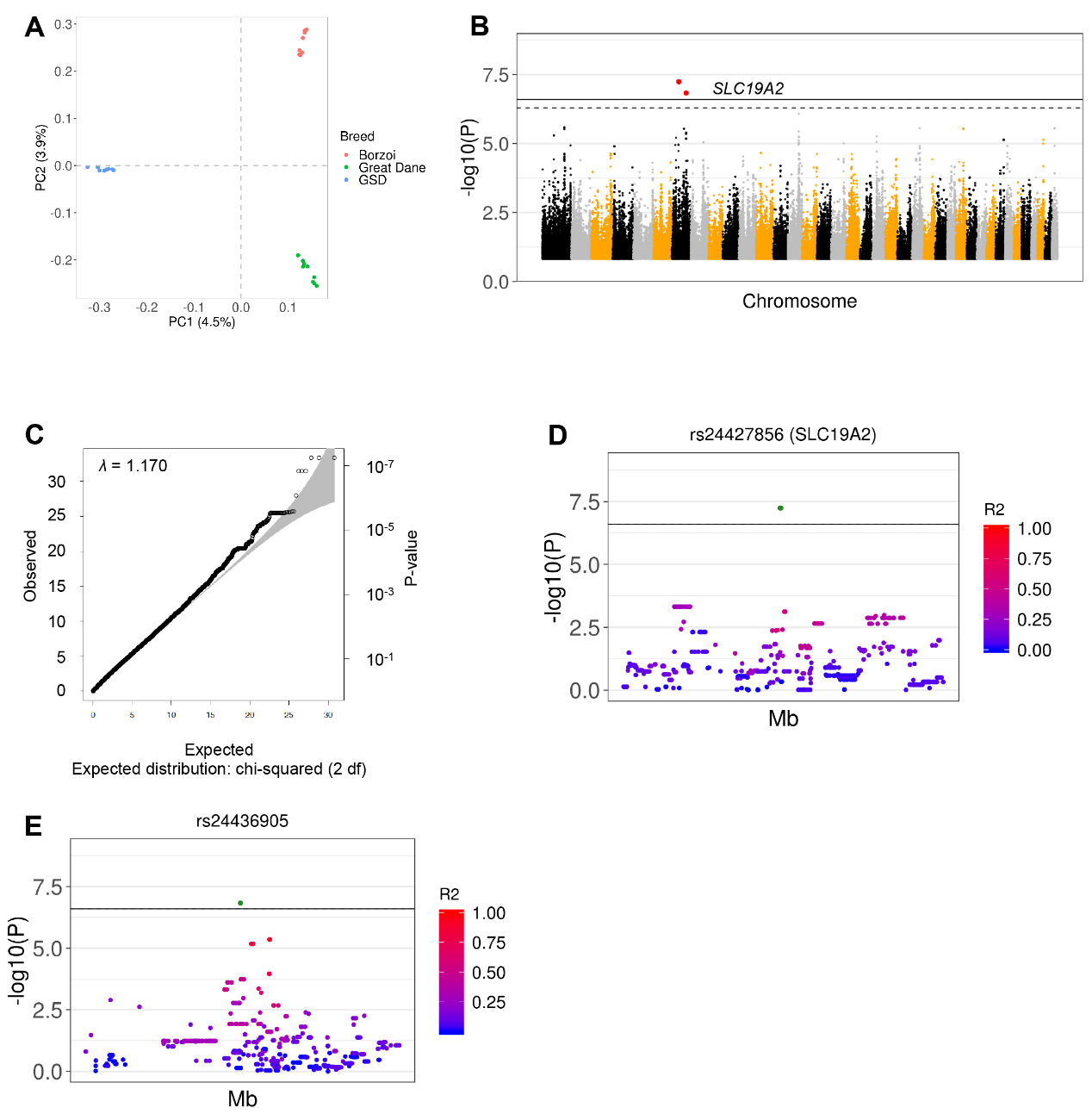


**Figure S3**: Identification of GDV-Associated SNPs based on GWAS from WGS Data in Borzoi, GSDs, and Great Danes Adjusting for GDV Family History. (A) Scatterplot showing the breed distribution using the two most informative Principal Components for the entire cohort, including all controls. (B) Manhattan plots after adjusting for sex, showing the two significant signals. (C) A qqplot showing the correlation between expected and observed test statistic distribution. The inflation factor showed some extent of population stratification (λ = 1.170), but not in the analysis non-adjusted for sex (λ = 1.018), where only the signal in the rs24436905 was detected. (D) Regional plot surrounding the significant SNP rs24427856 located in SLC19A2. The LD is shown as pairwise R^2^ between the top SNPs (rs24427856) and all other SNPs in the regions. (E) Regional plot surrounding the significant SNP rs24436905. The LD is shown as pairwise R^2^ between the top SNPs (rs24436905) and all other SNPs in the regions.


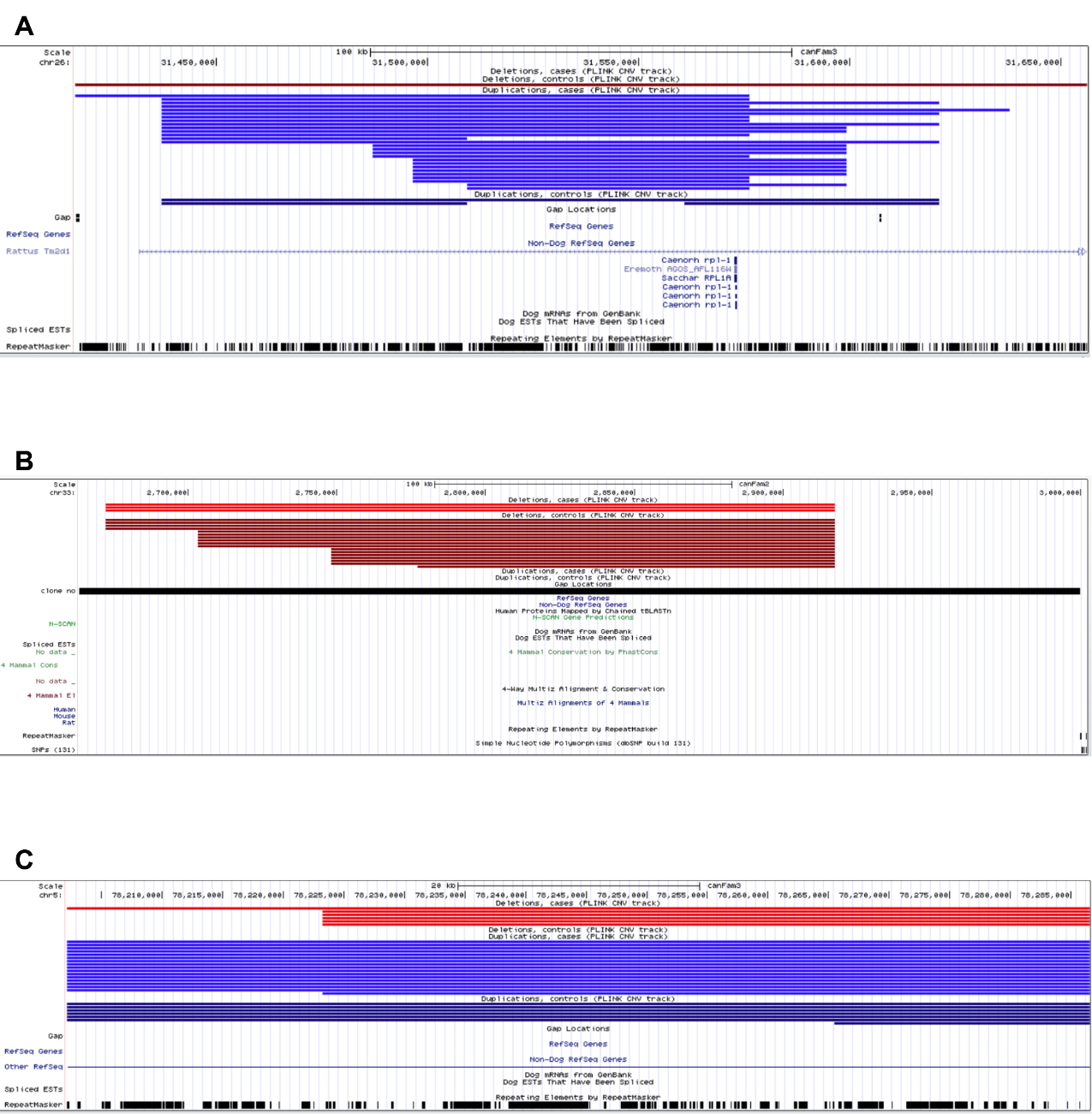


**Figure S4.** Relevant results obtained with the CNV analysis using the microarray data. (A) All breeds: region located in chr26:31,496,517-31,576,417 including a duplication with frequency significantly higher in AF than UF (p < 0.05). The finding was not confirmed when we use the SC cohort. (B) Borzoi, Great Dane and GSD: region located in chr33:2748039-2917476 including a deletion significantly more frequent in UF than AF (p = 0.001). The finding was confirmed when we used the SC cohort.
